# Effects of the reduction of surgical residents’ work hours and implications for surgical residency programs: a narrative review

**DOI:** 10.1186/1472-6920-14-S1-S14

**Published:** 2014-12-11

**Authors:** Mohammad H Jamal, Stephanie Wong, Thomas V Whalen

**Affiliations:** 1 Department of General Surgery, McGill University Health Centre, Montreal, Canada; 2 Department of Surgery, College of Medicine, Kuwait University, Kuwait City, Kuwait; 3 Department of Surgery, Lehigh Valley Health Network, Allentown, Pennsylvania, USA

## Abstract

**Background:**

The widespread implementation of resident work hour restrictions has led to significant alterations in surgical training and the postgraduate educational experience. We evaluated the experience of surgical residency programs as reflected in the literature from 2008 onward in order to summarize current challenges and identify key areas in need of further research.

**Methods:**

We searched MEDLINE and EMBASE for English-language articles published from January 2008 to December 2011 related to work hour restrictions in surgical residency programs, including those pertaining to personal well-being, education and training, patient care, and faculty experiences.

**Results:**

We retrieved 240 unique abstracts and included 24 studies in the current review. Of the 10 studies examining effects on operating room experience, 4 reported negative or mixed outcomes and 6 reported neutral outcomes, although non-compliance was demonstrated in 2 of these studies. Effects on surgical faculty perceptions were consistently reported as negative, while the effect on patient outcomes and professionalism were found to be neutral and unchanged.

**Conclusions:**

Further studies are needed to characterize operative experience at varying levels of training, particularly in the context of strict adherence to new work hours. Research that examines the effect of the work hour limitations on professionalism and non-operative educational activities, such as reading and simulation-based training, as well as sign-over practices, would also be of benefit.

## Background

The trend toward reductions in resident work hours constitutes one of the greatest challenges in modern surgical training. Traditionally, long duty hours during surgical residency were considered necessary to ensure both competency and patient safety in surgical training. Long work hours allowed residents to observe patients throughout their hospital stay and participate in pre-, intra-, and post-operative management [1]. The opportunity to observe a surgical procedure and see not only its effect on the presenting symptoms but also patterns of post-operative complications was considered fundamental to surgical education. The continuity of care that this enabled was also deemed an important mechanism for the prevention of medical error. Before the implementation of duty hour restrictions by the Accreditation Council for Graduate Medical Education (ACGME) in 2003, patient sign-over rounds were not viewed as a major component of patient care, since the same team was responsible for a given patient throughout his or her hospital stay. In surgical education, professionalism and professional values also focused on continuity of care, and shift work was eschewed in favour of values such as commitment of residents to their individual patients. As a result, the dictated work hours – which actively restricted the exposure and availability of residents to their patients – have traditionally been thought to deprive residents of opportunities to acquire the professional values and decision-making abilities of professional surgeons [2].

The changes in work hours implemented by the ACGME in 2003 were not well received by the surgical community. Further restrictions were implemented in July 2011, after the release of the Institute of Medicine (IOM) report *Resident Duty Hours: Enhancing Sleep*, *Supervision*, *and Safety*. The American College of Surgeons (ACS) responded to the report by emphasizing the adequacy of the 80-hour-per-week standard and the necessity of allowing flexible hours for senior residents [1]. The ACS also concluded that the new changes proposed by the IOM would be detrimental to residents’ training.

In an earlier study, we evaluated research on the effect of the ACGME work hour restrictions published in the first five years after their implementation [3]. We reported a positive effect on resident lifestyle and education but found a negative effect on surgical faculty, who, in opposition to the work hours, cited detrimental effects on patient care, continuity of care, and resident surgical competency. In this paper we evaluate the experience of surgical residency programs with the ACGME work hour restrictions as reflected in research published from the year 2008 onward to identify areas that need further research and recognize the challenges that confront surgical programs in this new era of graduate medical training.

## Methods

### Data sources

Using MEDLINE and EMBASE, we searched the English-language surgical literature published from January 2008 to December 2011 for research on the impact of surgical residents’ work hours on personal well-being, education and training, patient care, and faculty members.

### Search strategy

In MEDLINE, we first searched for articles about *residents* beginning with the controlled vocabulary terms (i.e., MeSH terms) “Physicians,” “Education, Medical, Graduate,” or “Internship and Residency,” as well as by searching the title and abstract fields for the keywords “resident,” “junior doctor,” or “junior physician.” We next searched for articles about *work hours* using the MeSH terms “Workload” or “Personnel Staffing and Scheduling” in addition to a keyword search using “work adj5 hour,” “doctor adj5 hour,” “physician adj5 hour,” “dut adj5 hour,” “resident adj5 hour,” “shift adj5 hour,” “workload,” “work schedule,” or “night float.” The following MeSH terms were used to search for *personal well-being*, *patient care*, and *learning*: “Work Schedule Tolerance,” “Sleep Disorders,” “Chronobiology,” “Chronobiology Disorders,” “Fatigue,” “Mental Fatigue,” “Patient Care,” “Treatment Outcome,” “Learning,” or “Professional Competence.” *Surgical* was searched using the MeSH terms “Specialities, Surgical,” or “Surgical Procedures, Operative,” as well as by using the subheading “surgery” and performing a keyword search for “surgical” or “surger.” The *residents*, *surgical*, and *work hours* searches were combined with the *personal well-being*, *patient care*, and *learning* searches with the Boolean operator “and.” We also performed an additional search combining our *residents*, *surgical*, and *work hours* searches with a keyword search for “education,” as well as with a subheading search for “education.” A similar search strategy was used in EMBASE.

### Selection of studies

**Figure 1:**
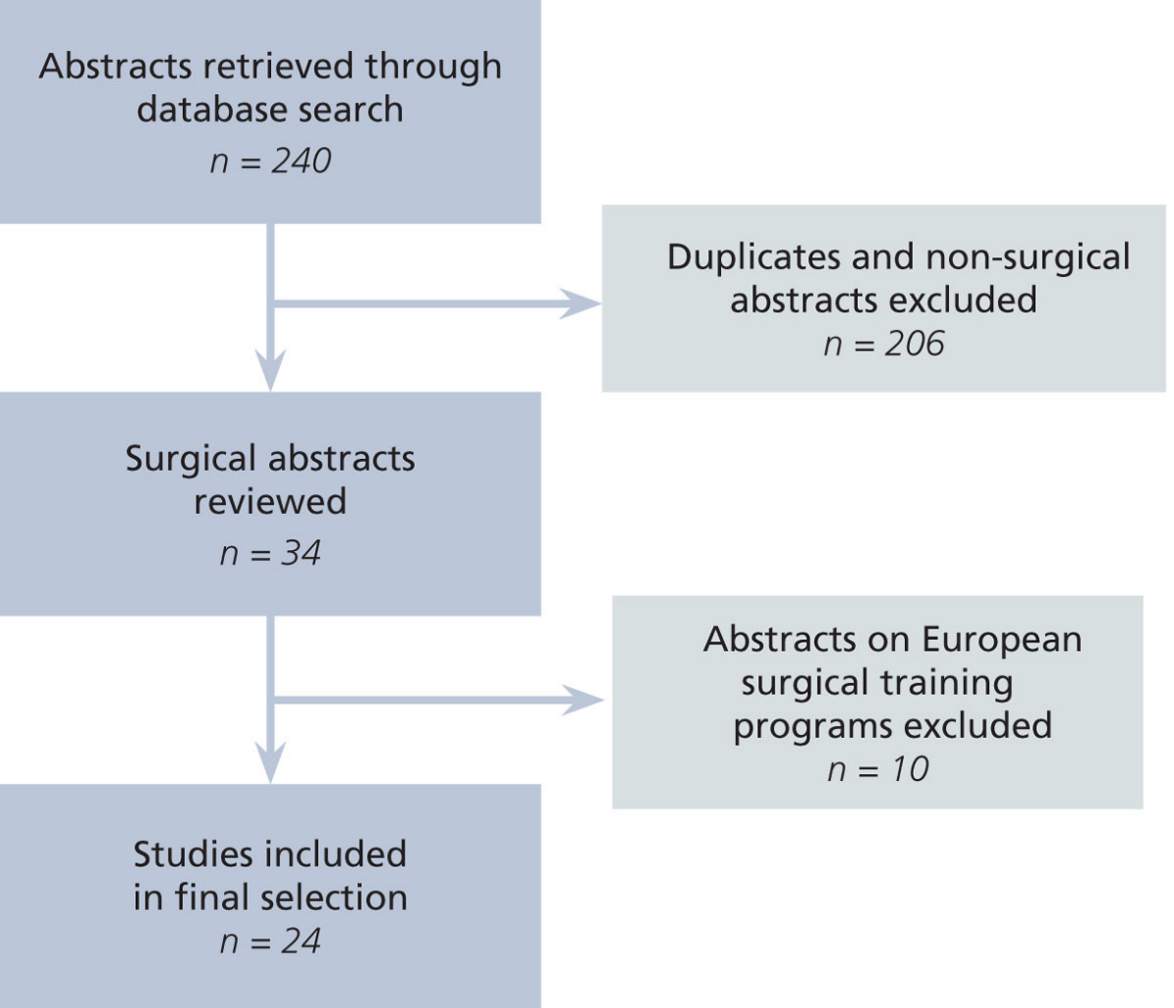
Flow chart of selection of studies for review

The MEDLINE and EMBASE search retrieved 240 unique abstracts. After the exclusion of non-surgical and non-relevant abstracts, 34 papers remained for detailed review. Ten pertained to European programs and were therefore excluded on the grounds that they did not examine similar reductions in work hours when compared to the ACGME restrictions that were uniformly applied and monitored in the United States. Therefore we reviewed 24 English-language papers evaluating the ACGME work hour restrictions in the United States (Figure 1) [4-27].

## Results

Of our final sample of 24 studies, 10 assessed residents’ operating room experience before and after the implementation of work hours restrictions [4-13], 5 evaluated patient care [14-18], 3 examined professionalism [19-21], 2 assessed the opinions of attending physicians [4,22], 2 assessed compliance with work hours [21,23], one examined residents’ attrition rates [24], 3 examined residents’ opinions [10,25,26], one examined the financial demands of the work hours restrictions [10], and one examined exam scores [8]. The additional file 1 summarizes the study findings.

The following analysis will focus on the impact of duty hour restrictions in the following areas: operating room experience, perspectives of surgical faculty, patient care, and professionalism.

### Impact on operating room experience

Ten studies that examined the effect of restricted hours on operating room experience were included in this review [4-13]. Each of the three studies that examined the total number of cases before and after the implementation of duty hour restrictions demonstrated a negative impact [6,7,11]. Hope and colleagues [9] surveyed residents and program directors and found that, before the work hour restrictions, 85% of cases were covered by residents, in contrast with only 60% of cases after the work hour restrictions. Picarella and colleagues [11] found that there was stability in the numbers of procedures performed by residents as primary surgeon and teaching surgeon, but a reduction in the number performed as first assistant. A large study by Simien and colleagues [12] examined the ACGME national and comparative reports of residents graduating in Urology, General Surgery, and Plastic Surgery. They noted no change in the volume of procedures performed by plastic surgery residents, an increase in procedures performed by Urology residents, and a reduction in vascular, plastic, and thoracic procedures performed by General Surgery residents. There was, however, an increase in the number of pancreatic, endocrine, and laparoscopic cases documented by General Surgery residents.

### Perspectives of surgical faculty

In our earlier review of the literature, we reported considerable dissatisfaction among surgical faculty with respect to the effects of the ACGME duty hour restrictions [3]. The report by Griner and colleagues [22] in our present sample of studies suggests that attending surgeons’ opinions of the work hour restrictions have remained negative. Comparing surgeons graduating in 2008 (that is, having trained entirely under the 80-hour work week) with those who had graduated before the restrictions, they found that 55% of attending surgeons had less trust in the 2008 graduates with respect to patient care and that 68% had less confidence in the ability of these residents to operate. Similarly, Antiel and colleagues [4] surveyed 719 program directors in Internal Medicine, Pediatrics, and General Surgery. With a response rate of 65%, the study noted a pronounced difference between Internal Medicine and General Surgery program directors’ perceptions of the work hour restrictions with respect to their impact on patient care: General Surgery program directors were significantly more likely to perceive that the ACGME restrictions would decrease residents’ ability to deliver high-quality and safe patient care.

### Impact on patient care

Initially, one of the main driving forces behind the restriction in residents’ work hours was the prospect of improvement in patient outcomes. Our group performed a meta-analysis examining the effect of work hour restrictions on surgical patients’ morbidity and mortality [16]. We analyzed 10 studies with 19 datasets, including 730 648 subjects in the mortality studies and 64 346 in the morbidity studies. We found no significant difference in morbidity and mortality after the work hour restrictions. The papers included in our meta-analysis were retrospective studies examining morbidity and mortality before and after such restrictions were implemented. Although none of the studies demonstrated a worsening of outcomes after the restrictions, this finding should be interpreted with caution.

### Impact on professionalism

Professional values continue to evolve in tandem with restrictions on work hours and their ensuing effect on continuity of care. Coverdill and colleagues [19] surveyed 15 General Surgery programs and performed 52 semi-structured interviews to examine the implications of restricted work hours for professionalism. Although the new professionalism dictates that residents work in teams, signing over patients from one shift to the next, Coverdill and colleagues [20] found that this practice was not followed in most of the programs they surveyed. Instead, residents tended to act in accordance with traditional professional values, continuing with their clinical duties despite the fact that they were surpassing work hour maximums. These researchers concluded that the evolution toward a “new professionalism” is stalled by a lack of emphasis on sign-over procedures. Similar results were reported by Szymczak and colleagues [21], who conducted ethnographic observations in Internal Medicine and General Surgery wards followed by interviews. These researchers concluded that residents had not yet migrated away from traditional professionalism, continuing to work beyond the reduced work hours to provide requisite care for patients, or to develop their own skills and knowledge. When interviewed, residents tended to present complex, nuanced reasoning to explain their non-compliance with the proposed hours.

## Discussion

Our review of the literature from 2008 onward assessing the impact of the ACGME duty hour restrictions on surgical training in the United States suggests results very similar to those of our review of the literature prior to 2008 [3]. At the same time, various research gaps mean that our picture of the impact of restricted work hours is incomplete.

### Resident education

At present, the effect on resident operating room experience appears neutral, although it may be too early to determine the exact impact, particularly in the context of non-compliance, as reported by two studies included in this review. Moreover, the current literature scarcely evaluates other educational aspects of surgical training, such as time spent in clinic or attendance at other academic activities such as morbidity and mortality meetings, grand rounds, and tumour board conferences. Although improving patient safety, rather than resident education, was the original impetus behind work hour restrictions, we might expect that the time freed up by such restrictions could be allocated to other educational activities such as reading. This potential benefit, however, has not been properly studied. The nuances of the impact of the ACGME restrictions on the service-to-education ratio for surgical residents therefore remain unclear.

Many residents now opt to extend their training through surgical fellowship programs; indeed, the numbers of applications for fellowship training positions have risen dramatically over the last decade, since the implementation of the work hour restrictions [28]. One possible solution to improve residency training without lengthening its duration may be to adopt a competency-based curriculum, keeping in mind that this might shorten the duration of training for some residents while lengthening it for others.

The shift from open surgeries toward laparoscopic procedures may also have an impact on residents’ comfort level with open surgery. Training that employs simulation technologies and skills labs could serve as a partial remedy to accelerate learning in the setting of reduced operative exposure [29]; however, this has not been studied in the literature with respect to open cases.

### Faculty perceptions

The current review is consistent with earlier reports on the dissatisfaction of surgical faculty with the reduction in work hours [3]. It is possible to conceive that work that can no longer be performed by residents under the restricted hours will be absorbed by faculty, to the detriment of their quality of life. In a retrospective cohort analysis published in 2009, Privette and colleagues [30] noted an increase in the supervision of residents by surgical faculty. Surgical faculty have also reported concerns about continuity of care, patient safety, and resident operating room experience, generally concluding that the effect of restrictions to resident work hours on these issues is negative [4,22].

### Patient care

Although the present review suggests that there has been no change to patient outcomes as the result of the restricted duty hours, this finding is difficult to assess. There may be covariables, such as increased faculty supervision, that explain why outcomes have not worsened despite the potentially adverse effects of, for example, more frequent handovers. The increased supervision noted by Privette and colleagues [30] is possibly the greatest change observed by faculty after the introduction of work hour restrictions, and was found to correlate with better patient outcomes in their study cohort. There is insufficient research, however, to determine the relative importance of reduced resident fatigue and increased faculty supervision, or the effect of increased supervision on the competency of surgical residents.

### Professionalism

The literature after 2008 still suggests that a new professionalism centred on good sign-out procedures and teamwork has not beem adopted and that residents continue to adhere to the old professionalism centred on patient “ownership.” Although the challenges faced by North American surgical programs are layered and complex, it is important for the surgical community to adapt to the new professionalism necessitated by the work hour restrictions. Surgical faculty’s dissatisfaction with the new hours can have the effect of subverting the restrictions, undermining the adoption of the new professionalism, and delaying improvements in processes to ensure patient safety. Particular emphasis should be placed on improving the efficiency of sign-out procedures and the safe transfer of patients to new residents under whom they will receive care. What seems clear, however, is that in many programs surgical residents are continuing to work beyond the proposed hours.

## Future directions

These results should be viewed in an international context. In 2000, the European parliament began to phase in limitations to resident work hours, which were reduced from 58 hours per week in 2004 to 48 hours in 2009. The response of European governments to these mandates has varied, such that countries have independently directed their own legislative efforts for resident work hours. To date, relatively little data exist on the effects of the European Working Time Directive, although a limited number of studies from Britain and Switzerland have reported decreased operative exposure [31-35], as well as negative perceptions on the part of residents with respect to surgical education and job satisfaction [35,36].

As the surgical community begins to accept that the reductions in duty hours are here to stay, further steps are required to prepare us for the future and a new era of professionalism. We must develop strategies to optimize the allocation of residents’ time, and structure residency education in a way that incorporates the best available evidence. Although the new duty hour requirements implemented by the ACGME in July 2011 offer greater flexibility for senior residents, the more rigid restrictions (including the maximum 16-hour work shift) placed on first-year residents have become a source of significant concern, and no objective data are available to assess their impact. In the wake of such change, we feel that further studies are warranted to examine the implications of these level-specific, graded regulations for resident education and surgical care. We support the recommendation of the ACS task force [1], in their response to the 2008 IOM report, to fund a multi-institutional study to identify optimal duty hours that achieve curriculum objectives, maintain continuity of care, and address team training efforts. Such a study should also examine the long-term effects of the ACGME duty hours and the impact they may have on readiness to enter surgical practice.

## Competing interests

The authors declare that they have no competing interests.

## Supplementary Material

Additional file 1 Summary of the study findings Click here for file

## Declarations

Resources and secretariat support for this project was provided by the Royal College.

This article has been published as part of *BMC Medical Education* Volume 14 Supplement 1, 2014: Resident duty hours across borders: an international perspective. The full contents of the supplement are available online at http://www.biomedcentral.com/bmcmededuc/supplements/14/S1. This article was submitted and peer reviewed in 2012. Final acceptance for publication as part of this supplement was in 2014.

Publication of this supplement was supported and funded by the Royal College of Physicians and Surgeons of Canada. The funding agency played no role in the design, in the collection, analysis and interpretation of data; in the writing of the manuscripts; and in the decision to submit the manuscript for publication. The articles have been through the journal's standard peer review process for supplements. The Supplement Editors declare that they have no competing interests.
