# Supplementary material for: Effects of the reduction of surgical residents’ work hours and implications for surgical residency programs: a narrative review
Source: BMC Med Educ. 2014 Dec 11;14(Suppl 1):S14. doi: 10.1186/1472-6920-14-S1-S14 (PMC4304271; doi:10.1186/1472-6920-14-S1-S14)
Supplement: Additional file 1 — Summary of the study findings [file 1472-6920-14-S1-S14-S1.pdf]

Study author, year	Positive/neutral outcomes	Negative outcomes	Effect on operating room experience	Program
Antiel et al. [4] 2011	None reported	Faculty less satisfied with continuity of care<br><br>Faculty perceives no effect on resident fatigue<br><br>Faculty perceives decreased ability to develop core competencies	Neutral	Internal medicine, pediatrics, general surgery
Browne et al. [14] 2009	None reported	Increase perioperative morbidity following treatment for hip fractures	Not assessed	Orthopedic surgery
Bruce et al. [5] 2010	None reported	None reported	Neutral	General surgery
Connors et al. [6] 2009	None reported	None reported	Negative (total cases)<br>Neutral (thoracic surgery)<br>Negative (cardiac cases)	Cardiothoracic surgery
Coverdill et al. [20] 2010	Residents believe they work longer than permitted in the name of professionalism<br><br>Residents see patient care as a team responsibility	None reported	Not assessed	General surgery
Coverdill et al. [19] 2011	None reported	Discrepancy between faculty and resident perceptions of work-hour restriction consequences for patient care	Not assessed	General surgery
Fairfax et al. [7] 2010	None reported	None reported	Negative (total cases)<br><br>Positive (gastrointestinal, skin/soft tissue and endocrine cases)<br><br>Negative (head/neck, vascular, pediatric, genitourinary, gynecological, endoscopic cases)	General surgery
Foley et al. [25] 2008	Positive perception by residents regarding preparedness for practice, case volume and complexity, duration of surgical training<br><br>Resident support for the work hour restrictions	None reported	None reported	General surgery
Froelich et al. [8] 2009	No effect on Orthopedic In-Training Examination scores	None reported	Neutral	Orthopedic surgery
Griner et al. [22] 2010	Faculty perceive neutral effect on resident attitude/interest, knowledge base, emotional stability, stamina, patient interactions, desire to be in operating room, satisfaction in career	Faculty perceive lower baseline work ethic, less-developed technical skill set, poor decision making/critical thinking skills, less patient “ownership”	Not assessed	General surgery

	choice	Faculty less trusting in patient care by residents<br><br>Faculty less confident in residents' ability to operate independently		
Helling et al. [15]<br>2010	No discenable effect on patient care (mortality and length of stay)	None reported	Not assessed	Trauma surgery
Hope et al. [9]<br>2011	None reported	None reported	Neutral	General surgery
Jamal et al. [16]<br>2012	No discernable effect on morbidity or mortality	None reported	Not assessed	General surgery, trauma surgery, orthopedic surgery, vascular surgery
Kamath et al. [27]<br>2011	None reported	Increased financial burden arising from need to hire physician extenders	Not assessed	Orthopedic surgery
Kashner et al. [10]<br>2010	Increased resident satisfaction with clinical environment and faculty	None reported	Neutral	Medicine, general surgery
Morrison et al. [17]<br>2009	Decreased patient mortality and length of stay	None reported	Not assessed	Trauma surgery
Picarella et al. [11]<br>2011	None reported	None reported	Negative (total cases)<br><br>Neutral (number of operations performed as primary surgeon)<br><br>Negative (number of operations performed as first assistant)	General surgery
Simien et al. [12]<br>2010	None reported	None reported	Neutral (plastic surgery residents)<br><br>Positive (urology residents)<br><br>Positive for pancreatic, endocrine, laparoscopic surgeries (general surgery residents)<br><br>Negative for vascular, plastic, thoracic surgeries (general surgery residents)	General surgery, urology, plastic surgery
Smith et al. [13]<br>2010	None reported	None reported	Neutral	Obstetrics/gynecology
Szymczak et al. [21]<br>2010	None reported	Non-compliance with work-hour regulations high<br><br>Ethical dilemmas encountered by residents high	Not assessed	General surgery
Tabrizian et al. [23]	Resident approval of work hour	Non-compliance with work-hour	Not assessed	General surgery

2011	regulations	regulations high		
Vaughn et al. [26]<br>2008	Perceived educational benefit by residents<br><br>Perceived mild benefit on patient care by residents<br><br>Perceived improvement in residents quality of life	Little perceived educational benefit by faculty<br><br>Perceived compromise in continuity of care	Not assessed	General surgery
Yaghoubian et al. [18]<br>2008	Decreased bile duct injury rate<br><br>Decreased overall complication rate	None reported	Not assessed	General surgery
Yeo et al. [24]<br>2010	None reported	Attrition rates remain high	Not assessed	General surgery
